# Behavioral Change Prediction from Physiological Signals Using Deep Learned Features

**DOI:** 10.3390/s22093468

**Published:** 2022-05-02

**Authors:** Giovanni Diraco, Pietro Siciliano, Alessandro Leone

**Affiliations:** National Research Council of Italy, IMM—Institute for Microelectronics and Microsystems, 73100 Lecce, Italy; pietro.siciliano@le.imm.cnr.it (P.S.); alessandro.leone@cnr.it (A.L.)

**Keywords:** behavioral change prediction, learned features, deep feature learning, handcrafted features, bidirectional long-short term memory, autoencoders, temporal convolutional neural network, clinical decision support system, multisensory stimulation therapy, physiological signals

## Abstract

Predicting change from multivariate time series has relevant applications ranging from the medical to engineering fields. Multisensory stimulation therapy in patients with dementia aims to change the patient’s behavioral state. For example, patients who exhibit a baseline of agitation may be paced to change their behavioral state to relaxed. This study aimed to predict changes in one’s behavioral state from the analysis of the physiological and neurovegetative parameters to support the therapist during the stimulation session. In order to extract valuable indicators for predicting changes, both handcrafted and learned features were evaluated and compared. The handcrafted features were defined starting from the CATCH22 feature collection, while the learned ones were extracted using a temporal convolutional network, and the behavioral state was predicted through bidirectional long short-term memory auto-encoder, operating jointly. From the comparison with the state of the art, the learned features-based approach exhibits superior performance with accuracy rates of up to 99.42% with a time window of 70 seconds and up to 98.44% with a time window of 10 seconds.

## 1. Introduction

In recent years, the detection and prediction of changes in time series data obtained from observations of a monitored system have become a relevant research topic in various fields [1,2,3]. In particular, change-point detection has attracted considerable interest in the medical and neurological fields, where the accurate determination of changes in physiological parameters is particularly critical [4,5].

Furthermore, change prediction is also important in supporting clinical decisions regarding the delivery of therapeutic interventions, such as in the case of the multisensory stimulation in dementia which was investigated in the MS-Lab (Multi Sensorial Stimulation Lab) project [6]. In MS-Lab, aiming to improve the efficacy of multisensory stimulation, a series of miniaturized non-invasive sensors located on the patient’s body were used to measure various neurovegetative parameters in real-time. A clinical decision support system (CDSS) was especially designed to find predictive patterns in multivariate neurovegetative time series (i.e., that anticipate behavioral reactions induced by therapy stimuli), and thus to provide useful hints to the therapist in selecting the most effective stimulation.

As it is well known, behavioral and non-behavioral reactions are induced by endogenous and exogenous stimuli. Behavioral reactions, such as expressing aggressiveness and facial emotions can be inhibited by voluntary control to some extent. On the other hand, non-behavioral reactions, as neurovegetative manifestations, are not under the influence of the cerebral cortex and are thus very difficult (if not impossible) to voluntarily control [7,8]. Furthermore, neurovegetative responses (i.e., physiological parameters), in terms of ergotropic and trophotropic reactions, can be considered as anticipatory of some behavioral reactions such as activation and relaxation [9,10,11].

The involuntary behavior is related to autonomic nervous system (ANS) functions such as sympathetic and parasympathetic activities: stressful or relaxing situations cause dynamic changes in ANS. More specifically, the sympathetic nervous system (SNS) dominates during stressful events, whereas the parasympathetic nervous system (PNS) dominates during resting behavior [12]. These concepts have been exploited in several studies to investigate the symptoms of stress, e.g., agitation, anger, fear and frustration, by measuring physiological neurovegetative parameters, since many of them are regulated by SNS and PNS, such as heart rate (HR), heart rate variability (HRV), respiration rate (RR), respiration amplitude (RA), galvanic skin response (GSR) and blood pressure (BP) [13,14]. In particular, various studies have shown [15,16] that skin temperature (ST) and GSR are indicators of stress level, i.e., high levels of stress are related to low levels of skin temperature due to the contraction of blood vessels, and low levels of skin resistance are due to an increase in body moisture.

Consequently, physiological neurovegetative parameters can be investigated as candidate indicators which may be able to anticipate the patient’s behavioral state when undergoing stimulation therapy to support decisions about the choice of the better stimulation to apply [6]. The typical situation is that of a patient with an agitated (AG) behavioral state who, subjected to multisensory stimulation, changes their behavioral state from AG to relaxed (RE). The hypothesis being tested in the MS-Lab project is that the AG-RE change in the behavioral state is somehow anticipated by the trend of physiological and in particular neurovegetative parameters. In that context, it is overwhelmingly important to detect early changes in physiological parameters suitable for predicting incipient changes in a patient’s behavioral state. The problem can be posed in terms of multivariate time series of physiological and neurovegetative parameters, and this involves identifying suitable features to highlight changes.

The watershed between methods for identifying changes in time series is undoubtedly represented by how features are obtained, which can be handcrafted or learned [17]. Most of the features reported in the literature are manually designed, i.e., handcrafted, paying attention to peculiar characteristics of the physiological parameters under consideration. The design of handcrafted features often requires finding a compromise between accuracy (ACC) and computational efficiency.

Healey and Picard [18] investigated the applicability of physiological signals from electrocardiogram (ECG), electromyogram (EMG), GSR and RA (i.e., the rib cage expansion) to determine the driver’s stress levels in a real-world scenario. The authors reported that three stress levels could be recognized with an overall ACC of 97.4% using statistical handcrafted features extracted from 5-minute data segments.

Handcrafted feature design is often associated with data fusion when dealing with multiple sensors, that is, the problem of how to integrate them to achieve better analysis results [19]. Zhang et al. [20] proposed a Bayesian network for the hierarchical merging of multi-sensor data, which differs from conventional approaches that integrate features such as a flat layer. Downstream of a two-stage process for selecting statistical features, the authors suggested an approach capable of autonomously learning the Bayesian network structure. The authors conducted the experiments using two public domain datasets for stress detection, including EMG, GSR, HR, RA, and BP signals, and so obtaining an ACC of 90.53%.

Among the various physiological and neurovegetative signals, the HRV analysis (i.e., R–R interval calculated from ECG peaks) effectively reflects the ANS regulation of cardiac activity [21]. Specifically, the high-frequency power of the HRV, i.e., from 0.15 Hz to 0.40 Hz, is associated with PNS activity, while the low-frequency power, i.e., in the 0.04–0.15 Hz band, is an indicator associated with the activity of the SNS. Wang et al. [22] investigated the use of HRV to distinguish physiological conditions under three different stress levels. The authors proposed a statistical feature selection algorithm based on a k-nearest neighbor classifier capable of exhibiting 88.28% ACC on a public domain dataset for assessing stress while driving.

In order to develop a prototype system for mental stress assessment, Chiang [23] combined various approaches such as single value decomposition (SVD), fuzzy theory, and associative Petri nets, to extract and analyze HRV from the ECG signal. The author extracted 12 features, both time-domain (statistical) and frequency-domain (power spectrum), from which the nine most representative ones were selected by using the information gain method. The reported results, obtained on a public domain dataset, showed an ACC of 95.10%.

Chen et al. [24] developed an automatic system to detect driving-related stress levels based on multi-channel physiological signals. Various features were extracted by the authors using wavelet decomposition, time, and spectral analysis, and combining sparse Bayesian learning (SBL) and principal component analysis (PCA) techniques to select the optimal feature set. Finally, they used Kernel-based classifiers to improve stress detection ACC, which was 89.70% on a publicly available dataset.

Zhang et al. [25] investigated the feasibility of recognizing different stress levels from the heterogeneous data of a physiological type (such as ECG, EMG, and GSR) and of a behavioral type, i.e., by using the reaction time. The authors employed visual and acoustic stressors to elicit stress in the subjects during the experiment, reporting a stress detection ACC of 92.36%.

Numerous methods have been devised to convert the time series of any complexity into feature sets that can represent the dynamic characteristics of the original time series [26]. The selection of features relevant to the problem under consideration was typically made manually, without a quantitative comparison between the various candidate features. Nevertheless, this handcrafted process left uncertainty about the optimality of the selected ensemble. For this reason, data-driven methods have recently been proposed that can make systematic comparisons between a large number of time series features. One of these approaches has been operationalized in the form of a Matlab^®^ toolbox with the name hctsa (highly comparative time-series analysis) [27].

Similarly, Lubba et al. [28] developed a data-driven framework called CATCH22 (22 Canonical Time-Series Characteristics), capable of distilling the most representative features from thousands of candidates extracted from a set of 93 different time series classification problems, including scientific, industrial, financial and medical applications. The authors implemented their framework in C language while providing wrappers for Python, R, and Matlab^®^.

The handcrafted features were obtained through a labor-intensive engineering process based on experience and a priori knowledge, marking the limits of current machine learning algorithms unable to extract all the juice contained in the data. In order to expand the applicability of machine learning algorithms, it is highly desirable to automate the feature extraction process, making the algorithms less dependent on feature engineering.

Feature learning, also known as representation learning or end-to-end learning, has recently established itself in the habit of deep neural networks (DNNs). Indeed, initially used to solve complex image classification and object recognition problems, DNNs have also proved helpful for extracting features regardless of the specific classification/regression problem on hand [29]. Furthermore, the deep feature learning process is intimately connected with unsupervised learning. In fact, the learning of features does not require labeled samples since the aspects relevant to the prediction problem under consideration (i.e., classification or regression) are somehow incorporated in the distribution of the input data. This is particularly true under the manifold hypotheses, according to which the elements of application interest are always associated with low dimensional regions (i.e., manifolds) included in the original data space [30].

According to the “greedy layer-wise unsupervised pre-training” paradigm [31], the feature hierarchy is learned one level at a time through the unsupervised learning of the transformation that connects one level to the next. In doing so, a layer of weights is added to the deep neural network at each iteration. Feature learning was applied by Wang and Guo [32] to the problem of recognizing the driver’s stress states. The authors proposed a two-stage model. Initially, the features were learned by deep learning based on multi-layer perception (MLP) auto-encoders (AEs) from physiological signals of ECG, GSR, HR, HRV and RA; subsequently, the stress states were recognized using the AdaBoost classifier. The reported results showed 90.09% ACC on a publicly available dataset.

Time series prediction, previously based mainly on analytical models, such as auto-regressive integrated moving average (ARIMA) [33], has recently been increasingly improved by deep learning models. In particular, the adaptation of the MLP to deal with time series is represented by the recurrent neural network (RNN), whose main characteristic is that the output of the hidden layer at the present instant is transferred to the hidden layer of the following time instant to preserve the time dependence of the data. However, in the presence of long-time dependencies, this transfer becomes difficult, raising the problem known as vanishing gradient in the back-propagation calculation [34]. To overcome such drawbacks, Hochreiter and Schmidhuber [35] proposed long short-term memory (LSTM), in which the internal structure of the hidden layers is more complicated by the presence of blocks equipped with the forget gate, input gate, and output gate. The distinctive aspect is that the memory cell state crosses the entire chain to selectively add or remove information through the intervention of the three gates.

Sundaresan et al. [36] proposed an LSTM-based DNN to classify mental stress from EEG scalp signals in young people with ASD. The results showed that mental stress states could be accurately assessed in adolescents with and without ASD, and adolescents with varying baseline levels of anxiety. The effectiveness of LSTM has been demonstrated, particularly for anomaly detection in time series with remarkable results [37,38,39]. Anomaly detection, in this case, is based on the application of the reconstruction error used as an anomaly score. An AE structure is often used to compress and reconstruct multi-dimensional input starting from non-anomalous training data. Indeed, AE cannot correctly reconstruct never-seen-before patterns of anomalous data, unlike previously seen patterns of non-anomalous data.

CNNs represent the most prominent example of deep learning exploitation for feature extraction, initially applied mainly to solve computer vision problems such as object recognition and classification [40,41,42], they were later also used for processing physiological multivariate time series [43,44]. However, CNNs are not born to manage temporal dependencies; therefore, to fill this gap, Bai et al. [45] proposed the temporal convolutional network (TCN), transposing the time-dependency problem from the RNN domain to the CNN one. TCNs proved superior to LSTMs in various application fields [46,47], also resulting to be computationally more efficiency [48].

Mou et al. [49] proposed a multimodal fusion model based on an attentional CNN-LSTM network that was able to fuse heterogeneous data coming from the driver’s eye (i.e., physiological signal), vehicle, and environment. In their approach, features were learned through CNN and LSTM, whereas different attention levels were assigned to features employing a self-attention mechanism. Data were segmented using synchronized sliding windows, selecting a window-size value equal to 5 seconds. The authors reported an accuracy performance of 95.5% with data fusion.

Nigam et al. [50] proposed an approach for automatically detecting stress from various physiological signals such as ECG, GSR, RA, body temperature (BT), and triaxial acceleration (TA). Their approach adopts handcrafted feature extraction with windows lasting 60 seconds, while stress detection is based on an LSTM network. The authors reported 98% accuracy performance on a freely accessible dataset.

With the purpose of detecting stress in car drivers, Zontone et al. [51] considered the physiological signals of GSR (taken from the driver’s hands) and ECG, from which they obtained various handcrafted statistical features. Then, considering feature windows lasting 15 seconds, the authors compared classical machine learning and deep learning approaches, reporting the best accuracy of 88.13% with LSTM.

The present study aimed to establish a comparison between handcrafted and learned features in predicting behavioral changes from physiological signals during multisensory stimulation therapy in dementia. The deep learning framework put together the benefits of TCN in feature extraction and of bidirectional long short-term memory (BLSTM) in change detection, resulting in increased computational efficiency and better detection performance. The change prediction of the patient’s behavioral status supports therapists in decision-making on selecting suitable stimulations. The study was carried out as part of the MS-Lab project [6], and the developed computational framework was integrated into the CDSS, currently undergoing clinical trials.

## 2. Materials and Methods

This section presents the algorithmic framework focusing on the behavioral change prediction from physiological multivariate time series by comparing approaches based on handcrafted and learned features. As explained in Section 2.2 and Section 2.3, the handcrafted feature approach uses the “Canonical Time-Series Characteristics” (CATCH22) collection of features and One-Class Support Vector Machine (OCSVM) to predict behavioral changes.

On the other hand, the learned approach relies on deep learning for feature extraction and change prediction. In this case, two learning approaches are compared. The first one adopts BLSTM-AE, presented in Section 2.4, for both feature learning and change prediction. Instead, the second learning approach uses TCN for feature learning (Section 2.5) and BLSTM-AE for change prediction, as explained in Section 2.6.

### 2.1. Experimental Setup and Data Acquisitions

**Figure 1:**
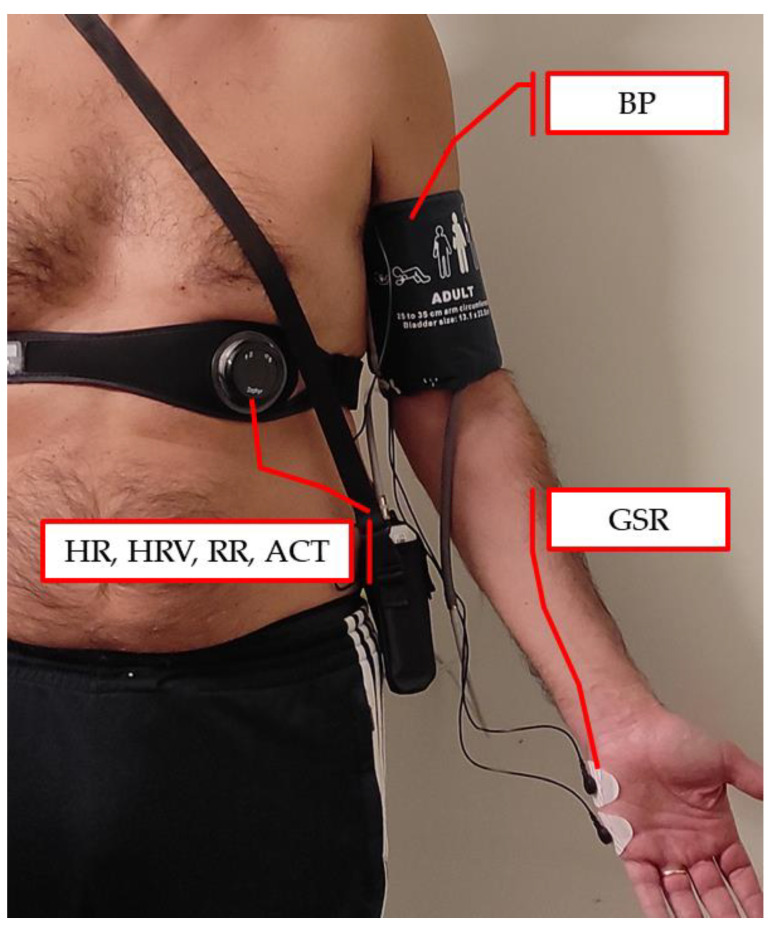
Multi-sensor setup for the acquisition of physiological and neurovegetative parameters.

The experimental protocol adopted in this study was approved by the Ethics Committee of the University of Salento (Lecce, Italy). The physiological signals of HR, RR, HRV, and activity level (ACT) were measured through chest strap Zephyr™ BH3 [52] worn by each subject during the stimulation session. The physiological signal GSR was measured using the MINDFIELD^®^ eSense [53] device with two electrodes attached to the outer side of the left-hand palm. BP was measured with a wearable cuff-based device manufactured by GIMA^®^ [54]. All the aforementioned sensors are shown in Figure 1 The physiological signals were collected in two different datasets, namely Dataset 1 (DS1) and Dataset 2 (DS2). In addition, an additional dataset DS2’ was also obtained starting from DS2 by suppressing the GSR signal. The main characteristics of the two datasets are summarized in Table 1 and described in detail below.

In the case of DS1, four patients were recruited from the nursing center of Casa Amata Srl (Taviano, Lecce, Italy) based on their degree of dementia severity, assessed through the Mini-Mental Statement Examination (MMSE) with a score lower than 10 points [6]. Four behavioral states were considered: active (AC), AG, apathetic (AP), and RE. Initially, each patient underwent neurological examination to establish the underlying behavioral state, i.e., AG or AP behavior. Two patients were enrolled with an underlying AG type clinical condition, and the other two with an AP underlying clinical condition.

Then, during the therapy session, each patient was subjected to multisensory stimulation lasting 7–13 minutes, after an initial period of equal duration in the absence of stimulation used as a baseline. The type of stimulation was chosen based on the patient’s underlying behavioral status and preferences. For example, in the case of a patient with AG behavior, stimulation will be selected to relax, i.e., to change the behavioral state from AG to RE. The stimulations used mainly consisted of exposure to video clips according to each patient’s preferences (e.g., dances and sounds of local folk, rock music bands, relaxing light colors and sounds).

HR, HRV, RR, and ACT parameters were acquired during the session. Instead, the BP and GSR parameters were not acquired as the measurement systems (i.e., electrodes attached to the palm and a cuff in the arm) were not adequate for the clinical conditions of some patients (especially for those with AG behavior). The therapist manually annotated the behavioral states manifested by each patient.

Regarding the DS2 dataset, to further validate the algorithmic framework with also additional physiological signals, i.e., the GSR and BP, the dataset was collected by involving five healthy volunteers. The participants were exposed to different stimulation scenarios to elicit the four behavioral states, namely the AC, AG, RE, and AP. 

Specifically, for the AC and AG behaviors, the participants were asked to watch short video clips selected from the FilmStim database [55] to elicit specific emotions as follows: AC—amusement, sadness, tenderness; and AG—anger, fear, disgust. In the case of the AP behavioral state, the elicited emotion was boredom by exposing the volunteers to the repetitive task of performing orally simple arithmetic operations displayed on the screen.

Finally, the RE behavior was simulated using the relaxation VR application developed by TRIPP, Inc. [56] for the VR viewer Oculus Quest 2 [57]. The exposure to stimulations lasted from 15 to 60 minutes, and the same volunteer signaled the beginning of the new behavioral state. Then, from the beginning of the new behavioral state, the neurovegetative signals were extracted for a duration equal to the dataset DS1, i.e., approximately 7–13 minutes.

### 2.2. Data Preprocessing and Handcrafted Features

All signals provided by the BH3 [52] and eSense [53] devices were sufficiently clean, so filtering was unnecessary. The BH3 device provided the HR, HRV, RR, and ACT signals at one sample per second, whereas the eSense device sampled the GSR signal with a sample rate of 5 Hz, so it was necessary to down-sample the GSR signal at 1 Hz.

In order to have balanced datasets, the duration of the acquired stimulations was standardized to 7 minutes each. Physiological signals were treated as multivariate time series by extracting sliding windows lasting 10–70 seconds at a one-second step. Thus, the processed time -series samples varied from 351 (window duration of 70 s) to 411 (window duration of 10 s) for each behavioral state, obtaining a total amount of samples per subject ranging from 1364 to 1604 in the case of DS1, and from 1023 to 1203 in the case of DS2.

In order to better evaluate the handcrafted features compared to the learned ones, the statistical features defined in the previous study [58] were extended by extracting the 22 features suggested by Lubba et al. [28]. This collection of features, called by the authors CATCH22 and summarized in Table 2, is the result of a selection among 5000 candidate features, carried out by evaluating the classification performance of 93 different time series.

Let $d_{S}$ be the stimulation duration (in seconds), and let $c_{W}\in \left\{10,\;15,\;20,\;30,\;40,\;50,\;60,\;70\right\}$ be the window duration (in seconds), the number of sampled windows is given by $n_{W}=d_{S}-c_{W}$, and the feature extraction process can be defined as the following map function

(1) $$CATCH22\;:\;ts_{V}\in {\mathbb{R}}^{4\;n_{W}\times n_{C}\;c_{W}}\to ts_{F}\in {\mathbb{R}}^{4\;n_{W}\times n_{C}\;n_{F}}$$

where $n_{C}\in \left\{4,\;6\right\}$ is the number of collected physiological signals; $n_{F}=22$ is the number of extracted features; $ts_{V}$ is the time-series matrix; and $ts_{F}$ is the corresponding feature matrix.

### 2.3. One-Class Support Vector Machine

Once the handcrafted features are defined, they can be exploited for the detection of behavioral change. The behavioral change detection can be posed as a one-class classification problem where the target (or normal) class corresponds to the behavioral state observed prior to the stimulation administration, also called baseline state. As is well known, the one-class classification is characterized by a sufficiently large number of samples belonging to the target class, while the samples belonging to classes not of interest (outliers) are absent or very few. Such a condition is naturally satisfied by the application under consideration, i.e., behavioral-state change detection, since it is the therapist that determines the initiation of the multisensory stimulation. Thus, the classifier training occurs during the baseline state, and it ends when a stimulation is applied. The classic approach (i.e., not based on deep learning) to the one-class classification is represented by OCSVM, whose classic formulation enables using hyperplanes to isolate the target class samples from outliers that are assumed to fall on the plane through the origin [60]. Hence, the OCSVM algorithm maps data points of the feature space ($ts_{F}$) into the Kernel space to separate them with the maximum margin, assigning the value +1 to points of the target class and −1 to the other points. 

Let $u\in {\mathbb{R}}^{N}$ be the normal vector of the hyperplane separating the target class from the origin, let $z_{i}\in {\mathbb{R}}^{N}$ be the i-th row of $ts_{F}$ (i=1,…,M), let $\xi \in {\mathbb{R}}^{M}$ be slack variables that penalize the outliers, let ρ∈ℝ be the maximum separation distance of the hyperplane from the origin, and let $\nu \in ]0,1]$ be the upper bound on the percentage of outliers; hence, the normal vector u is given by solving the following maximization problem

(2) $$\underset{∥u∥,\xi ,\rho }{\max}\frac{1}{2}u^{2}+\frac{1}{\nu \;M}\overset{M}{\underset{i=1}{\sum }}\xi_{i}-\rho \;\mathrm{subject}\;\mathrm{to}\;\forall i=1,\ldots ,M:u\cdot z_{i}^{T}\geq \rho -\xi_{i},\;\xi_{i}>0.$$

**Figure 2:**
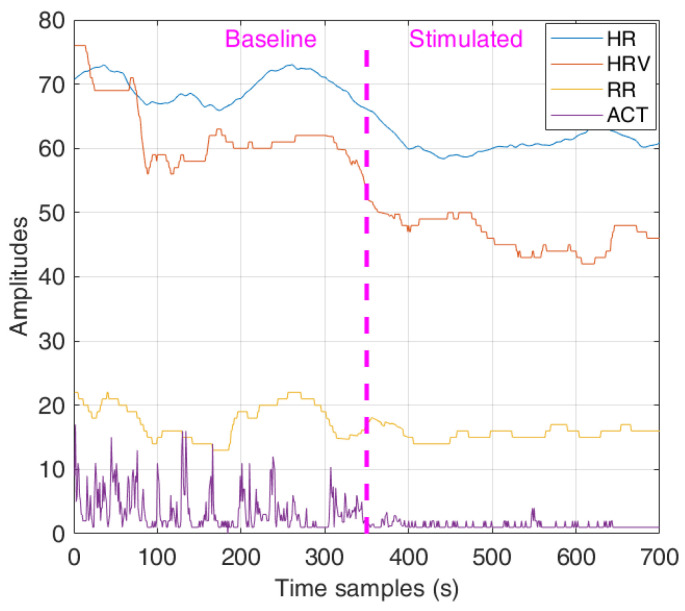
Physiological signals in baseline (AG) and stimulated (RE) behavioral states.

Considering the physiological signals measured during a multisensory therapy session, as shown in Figure 2, the baseline physiological signals are used to train the OCSVM model. After that, during the stimulation phase, changes in the physiological parameter can be detected through model testing, predicting a behavioral state change (BSC), e.g., from AG to RE in the example reported in the previously mentioned figure.

### 2.4. Bidirectional Long Short-Term Memory Autoencoders

To analyze the prediction of behavioral change based on BLSTM-AE, it is first necessary to introduce the concept of LSTM and then that of AE. Let $x_{i}\in {\mathbb{R}}^{N}$ be an input time-series data (i.e., the i-th row of $ts_{V}$), let $W_{k},R_{k}\in {\mathbb{R}}^{M\times N}$ $\left(\forall k\in \left\{Ι,\iota ,f,o\right\}\right)$ be weight matrices, let $b_{k}\in {\mathbb{R}}^{M}$ $\left(\forall k\in \left\{Ι,\iota ,f,o\right\}\right)$ be bias vectors, an LSTM memory cell at time step i is defined by its input ${Ι}_{i}$, its state $c_{i}$, its output $O_{i}$ and its gates $\iota_{i},f_{i},o_{i}$ (input gate, forget gate, and output gate, respectively), and hence its transition equations are given as follows

(3) $$\begin{array}{l} {Ι}_{i}=h\left(W_{Ι}x_{i}+R_{Ι}O_{i-1}+b_{Ι}\right)\\ \iota_{i}=\sigma \left(W_{\iota }x_{i}+R_{\iota }O_{i-1}+b_{\iota }+p_{\iota }\circ c_{i-1}\right)\;\\ f_{i}=\sigma \left(W_{f}x_{i}+R_{f}O_{i-1}+b_{f}+p_{f}\circ c_{i-1}\right)\;\\ o_{i}=\sigma \left(W_{o}x_{i}+R_{o}O_{i-1}+b_{o}+p_{o}\circ c_{i}\right)\\ c_{i}=\iota_{i}\circ {Ι}_{i}+f_{i}\circ c_{i-1}\\ O_{i}=o_{i}\circ h\left(c_{i}\right)\; \end{array}$$

where $\left(p_{\iota },p_{f},p_{o}\right)$ are three peephole connections scaling the gates with the cell state, $\sigma \left(\cdot \right)$ is the sigmoid activation function, $h\left(\cdot \right)$ is the hyperbolic tangent activation function, and ∘ denotes the Hadamard product.

**Figure 3:**
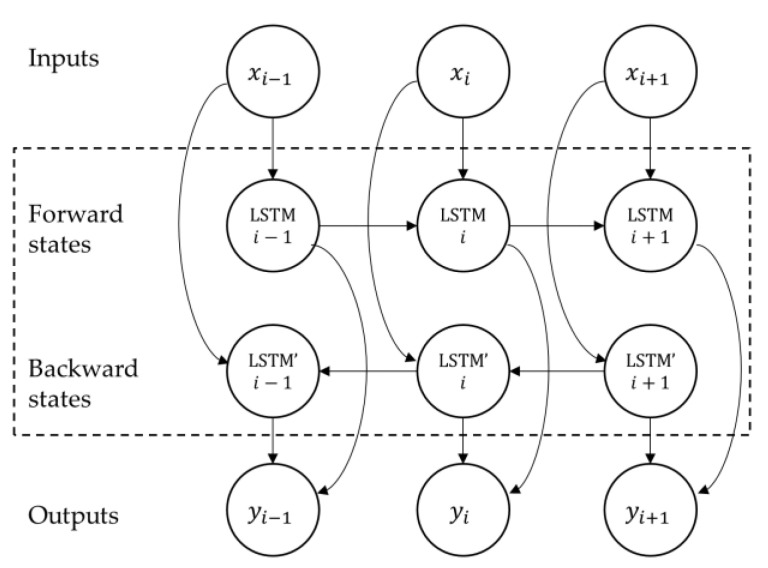
Diagram of a BLSTM layer.

The structure of the LSTM memory cell described above allows the network to access long time-series sequences in both backward and forward directions (within the same time window). The general structure of such a BLSTM network is shown in Figure 3.

In this paper, the BSC prediction using feature learning was accomplished via AE, in which both the encoder and decoder networks are based on BLSTM. An AE is an unsupervised neural network essentially consisting of an input layer, an encoder neural network, a decoder neural network, and an output layer. Once compressed by the encoding, the data provided as input are represented in the so-called latent space. Then, the decoder decompresses such a latent representation, trying to reconstruct the input data into output.

More specifically, let $z_{i}\in {\mathbb{R}}^{N}$ (with i=1,…,M) be the time series provided as input to the AE network, let $E\left(z_{i}\right)\in {\mathbb{R}}^{N^{\prime }}$(with $N^{\prime }<N$) be the encoded representation provided by the encoder network, let ${\hat{z}}_{i}=D\left(E\left(z_{i}\right)\right)\in {\mathbb{R}}^{N}$ be the reconstructed input provided by the decoder, and the AE training consists of minimizing the reconstruction error

(4) $$\mathrm{RE}\left(z,\hat{z}\right)=\frac{1}{2}\sum_{i=1}^{M}{∥z_{i}∥-∥{\hat{z}}_{i}∥}^{2}$$

which is backpropagated through the network to update the weights.

The effectiveness of the AE in learning features lies in constraining the latent space to be smaller than the input ($N^{\prime }<N$), which forces the neural network to learn the most salient features of the time series data

$ts_{V}$.

**Figure 4:**
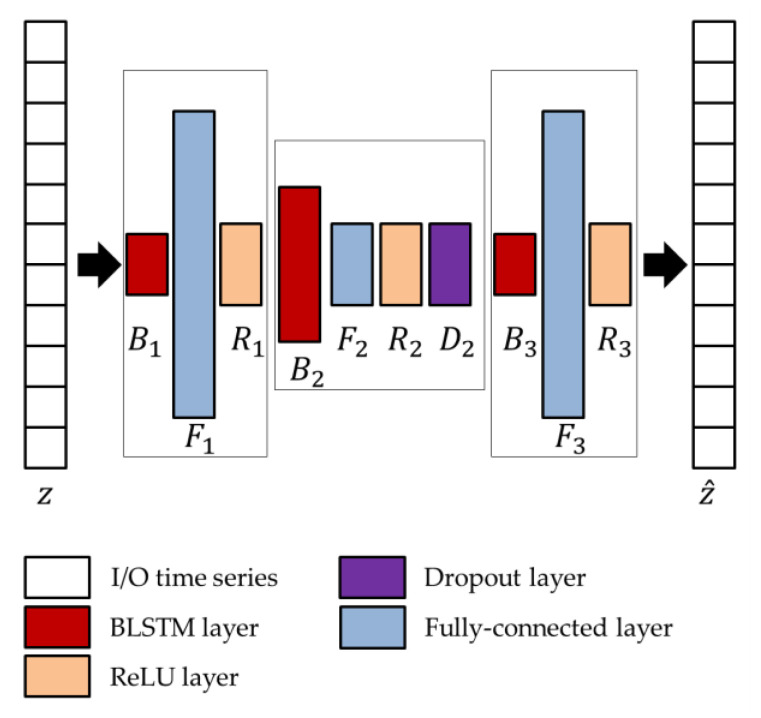
Architecture of the BLSTM-AE network.

The network parameters of the BLSTM-AE architecture, whose overview is shown in Figure 4, are optimized using the genetic approach presented by Diraco et al. in [61]. For this purpose, a variable number of blocks is considered as ranging from 3 to 5, with two external and one more internal, each block consisting of BLSTM and fully connected, in addition to rectified linear unit (ReLU) and dropout layers, where the last two layers are optional. At the end of the optimization process, the obtained architecture is composed of three blocks, among which the first and last only consist of the BLSTM ($B_{1}$ and $B_{3}$) and fully connected ($F_{1}$ and $F_{3}$) layers, while the central one includes all layers ($B_{2},\;F_{2},\;R_{2},\;D_{2}$). Regarding the network parameters, the number of hidden units $N_{k}$, the output dimensions $F_{k}$, and the dropping out probability $D_{2}$ are provided in Table 3.

### 2.5. Temporal Convolutional Network

In order to increase the representational power of learned features also in situations of temporal dependencies that go beyond a single observation window, the use of a supervised pretrained network based on temporal convolution (i.e., TCN) was investigated in combination with the previously described unsupervised bidirectional long short-term memory autoencoder (BLSTM-AE) network. In addition, to not jeopardize the basic unsupervised structure of the BLSTM-AE approach, the pre-training of the TCN was conducted on the DS2 dataset, which was different from that used for validation, i.e., the DS1 dataset.

TCN networks [45] are convolutional networks specifically designed to process time series, which are similar to LSTM networks but with even better performance. The main feature of TCN networks is implementing a dilated causal convolution, i.e., it only involves values temporally prior to the current one. This allows the network to capture long-term patterns, increasing the receptive field without resorting to pooling and thus, avoiding the loss of resolution [62].

Given the input sequence $x\in {\mathbb{R}}^{N}$, the dilation factor d, the convolutional kernel g of size S∈ℕ (with N>S>d), thus the dilated causal convolution (DCC) with dilation factor d at the time instant i is defined as follows

(5) $${\mathrm{DCC}}_{d}\left(x,g\right)\left(i\right)=\overset{S-1}{\underset{j=0}{\sum }}g\left(j\right)x_{i-dj}$$

that for d=1 corresponds to classical convolution. By exponentially increasing the dilation factor at each layer, it is possible to obtain a wider receptive field. As such, considering a total amount of K layers, the size D of the receptive field of the network is given by

(6) $$D=\left(S-1\right)\left(2^{K}-1\right)+1$$

**Figure 5:**
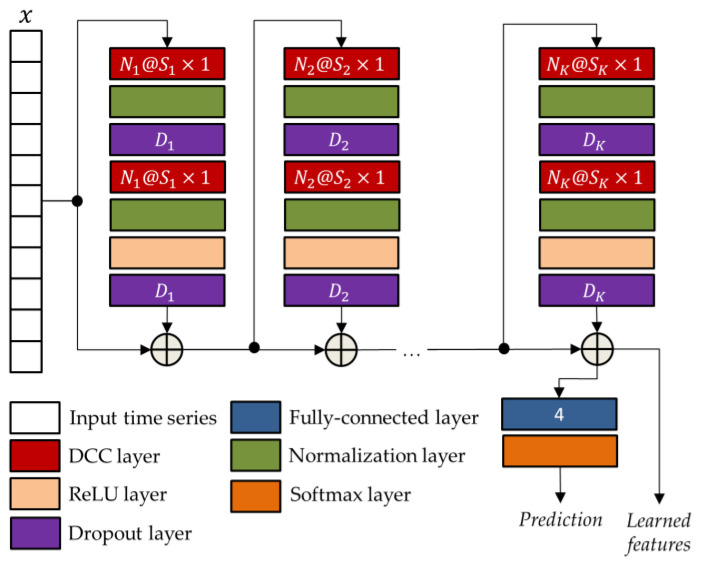
The general TCN architecture with K residual blocks.

The general TCN architecture, provided in Figure 5, has a modular structure based on K residual blocks, each including two DCC with an equal dilation factor, depth, and size. Suck blocks are characterized by residual connections which, as suggested by He et al. [63], improve the performance of deep architectures by adding the block input to its output.

As for the BLSTM-AE network, also in the case of the TCN network, the parameters were optimized using the genetic approach presented in [61]. The corresponding optimized parameters, i.e., the numbers of convolutional filters $N_{k}$, the filter sizes $S_{k}$, and the drop out percentages $D_{k}$, are reported in Table 4.

### 2.6. Joint Temporal Convolutional Network and Bidirectional Long Short-Term Memory Autoencoders

**Figure 6:**
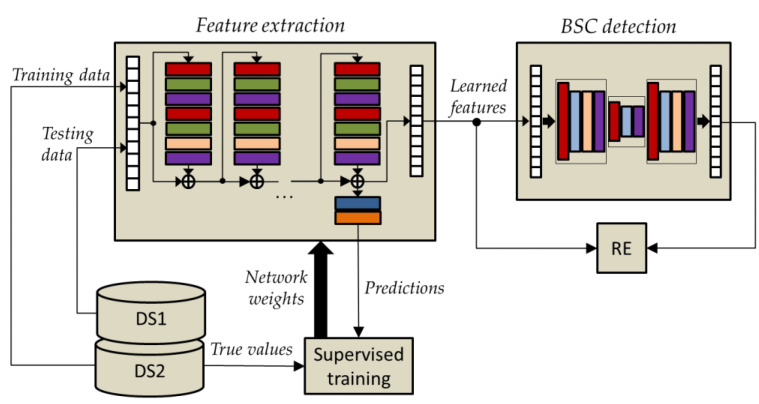
General overview of the joined architecture including the TCN and BLSTM-AE networks.

As already mentioned, the two networks TCN and BLSTM-AE are put together in order to increase the representation power of the learned features. In the BLSTM-AE TCN joint architecture, the TCN network plays the role of feature extraction, while the BLSTM-AE network plays the role of detecting BSC. As shown in Figure 6, the TCN is pre-trained by using time-series data from DS2, whereas DS1 time-series data are solely used for testing. This distinction enables maintaining unsupervised operation during the testing phase.

It is important to note that the TCN pretraining is performed by simulating the behavioral states AC, AG, RE, and AP, involving healthy volunteers, i.e., whose physiological parameters are collected in the DS2’ dataset. Instead, in the testing phase, the joined networks operate in unsupervised mode since activations (i.e., learned features) extracted from the pre-trained TCN are supplied as input to the BLSTM-AE, which naturally operates in an unsupervised manner, and then the RE is estimated comparing learned features and reconstructed ones using Equation (4). The workflow of the pretraining/training and testing steps of the TCN and BLSTM-AE networks can be summarized as follows:

(1) Supervised pretraining (with four classes: AC, AG, AP, RE) of the TCN-based feature extraction network using the DS2 dataset;
(2) Refining the TCN network using the DS1 dataset under baseline conditions (i.e., without multisensory stimulation);
(3) Testing (feature learning) of the TCN network using the DS1 dataset under baseline conditions (i.e., without multisensory stimulation);
(4) Training of the BLSTM-AE network using learned features from point 3) (baseline conditions);
(5) Testing (feature learning) of the TCN network using the DS1 dataset under stimulation conditions (i.e., with multisensory stimulation);
(6) Testing (BSC detection) of the BLSTM-AE network using learned features from point 5) (stimulated conditions).

**Figure 7:**
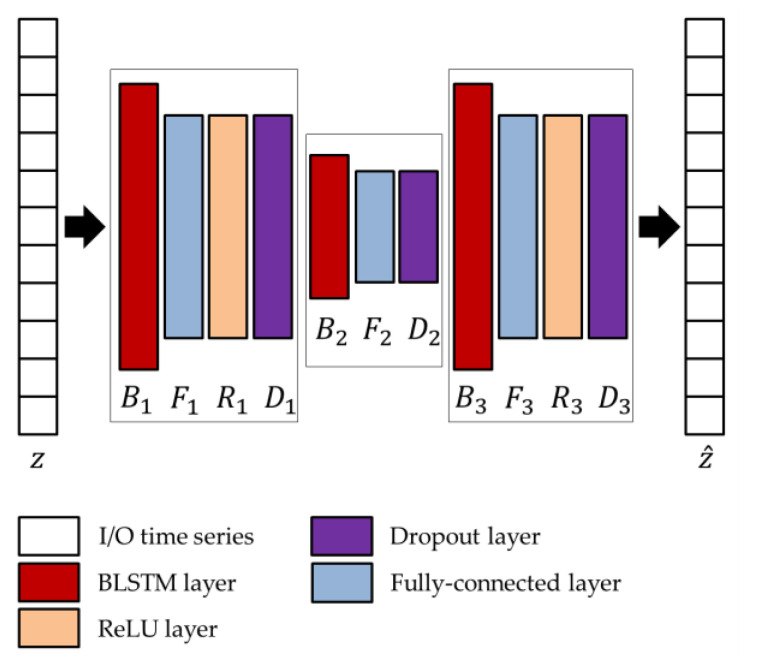
Architecture of the BLSTM-AE network adopted in conjunction with the TCN.

In the joint architecture, the parameters of the BLSTM-AE network are optimized again on the basis of the activations extracted from the TCN network, and by following the approach presented in Diraco et al. [61]. The optimized architecture is provided in Figure 7, and optimized network parameters, i.e., number of hidden units $B_{k}$, output size $F_{k}$, and dropping out probability $D_{k}$, are reported in Table 5.

## 3. Results

A performance comparison of the three approaches is provided in Table 6. As can be seen, although generally the ACC percentages decrease with the window (WD) durations, this trend is much less pronounced in the case of the BLSTM-AE TCN approach. The OCSVM approach based on handcrafted features has been evaluated in correspondence with windows of duration equal to or greater than 15 s, since not all the features of the CATCH22 collection are defined for windows of shorter duration.

The ACC is defined in terms of true positives (TPs), true negatives (TNs), false positives (FPs), and false negatives (FNs) as follows

(7) $$\mathrm{ACC}=\frac{\mathrm{TP}+\mathrm{TN}}{\mathrm{TP}+\mathrm{TN}+\mathrm{FP}+\mathrm{FN}}$$

TP, TN, FP, and FN refer to change predictions, and more specifically, TP denotes the changed states correctly predicted as changed; TN denotes unchanged states correctly predicted as unchanged; FP denotes unchanged states incorrectly predicted as changed, and FN denotes changed states incorrectly predicted as unchanged. 

**Figure 8:**
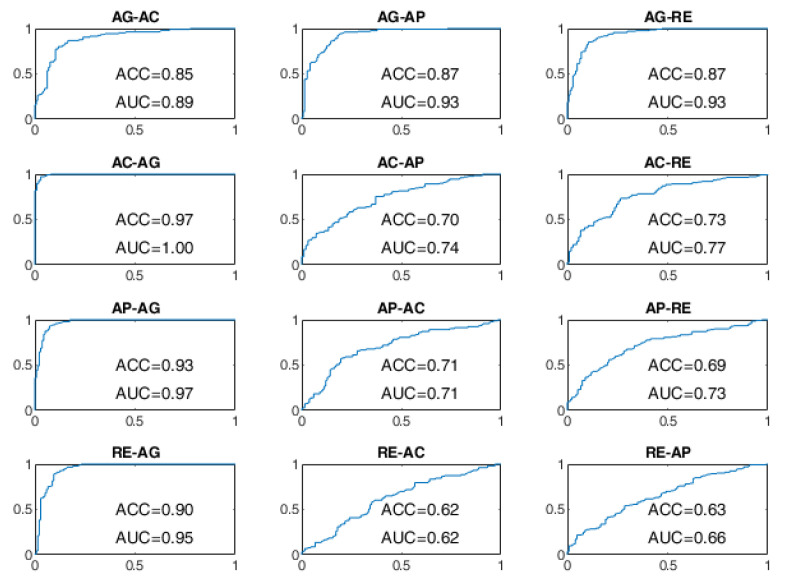
ROC curves of the OCSVM approach on the DS1 dataset for WD = 15 s.

**Figure 9:**
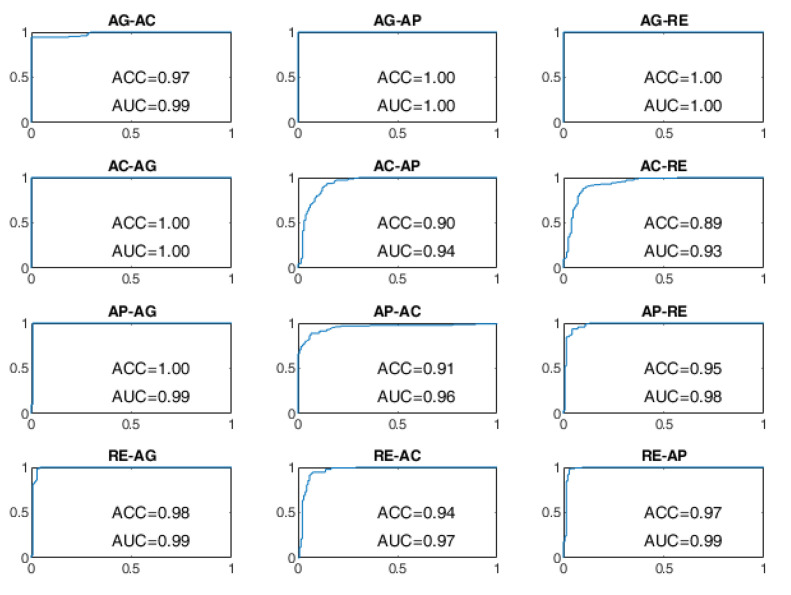
ROC curves of the OCSVM approach on the DS1 dataset for WD = 70 s.

**Figure 10:**
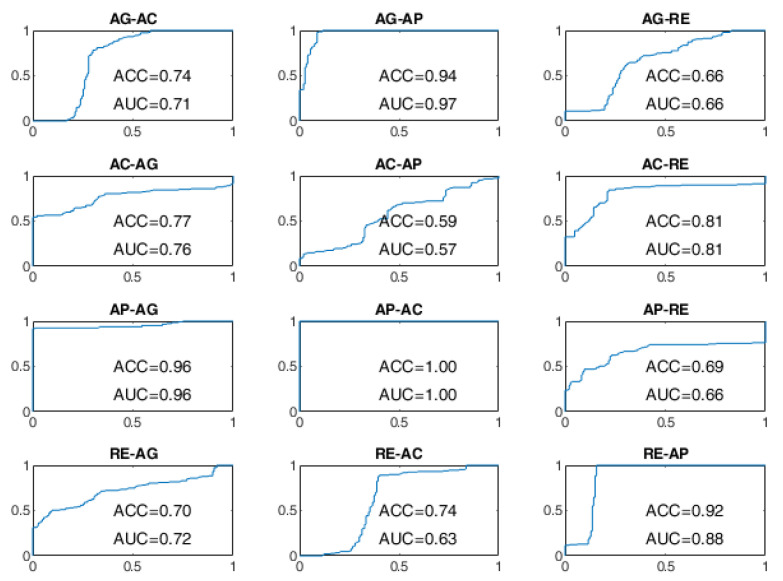
ROC curves of the BLSTM-AE approach on the DS1 dataset for WD = 15 s.

**Figure 11:**
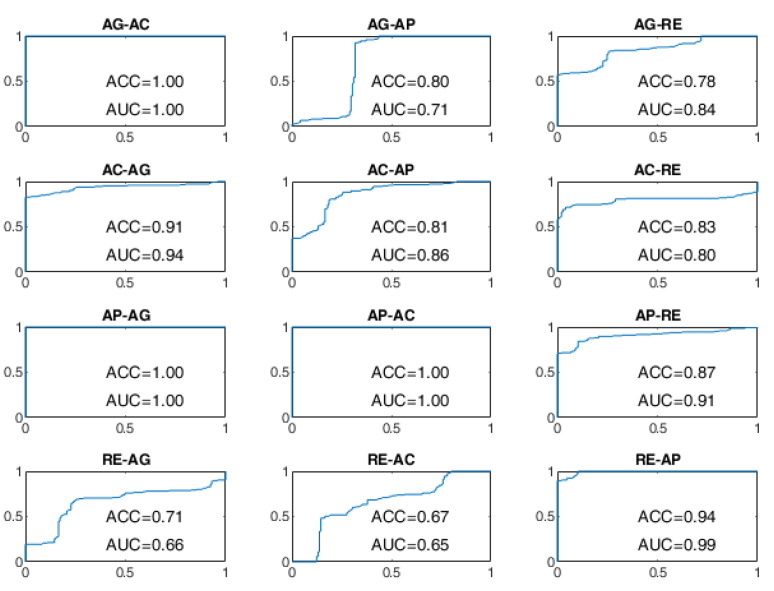
ROC curves of the BLSTM-AE approach on the DS1 dataset for WD = 70 s.

**Figure 12:**
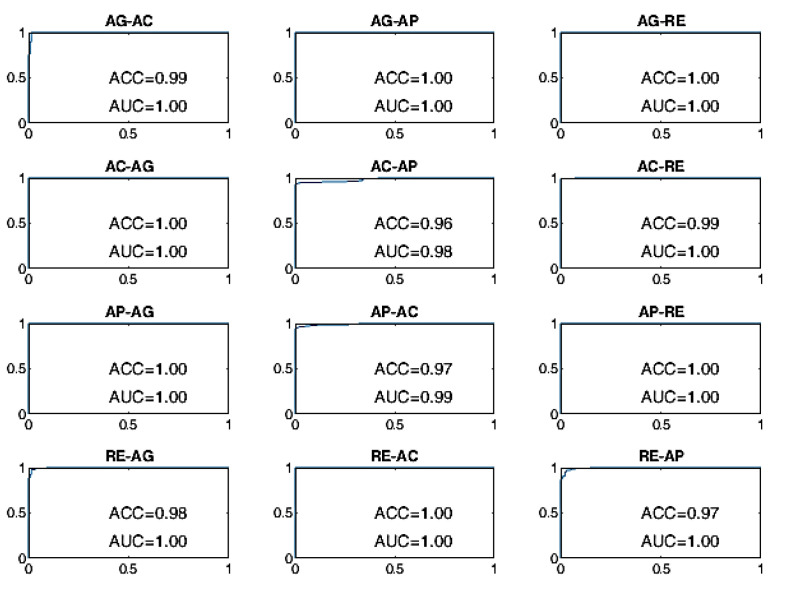
ROC curves of the BLSTM-AE TCN approach on the DS1 dataset for WD = 15 s.

**Figure 13:**
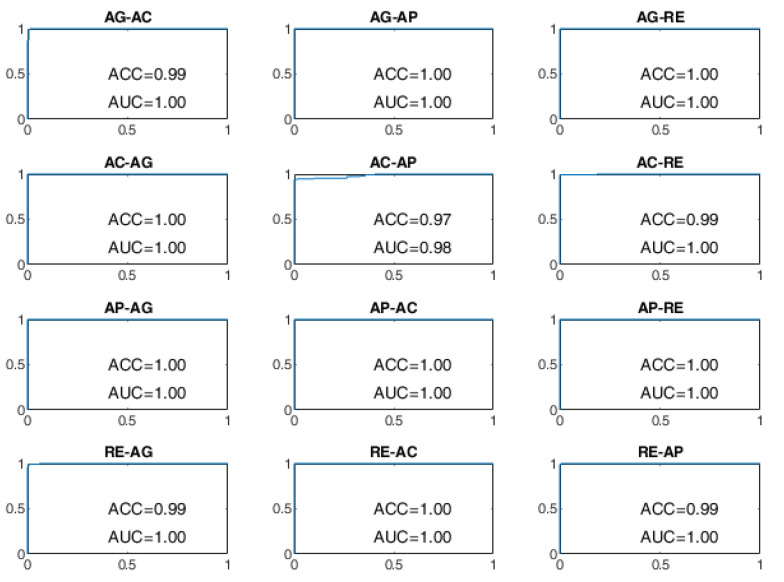
ROC curves of the BLSTM-AE TCN approach on the DS1 dataset for WD = 70 s.

All BSCs are considered from the baseline behavioral state to a different behavioral state manifested after the stimulation. For example, the AG–AC change indicates the transition from the baseline state of AG to the stimulated state of AC after the administration of sensory stimulation. The receiver operating characteristic (ROC) curves of the BSP achieved with the evaluated approaches are reported from Figure 8, Figure 9, Figure 10, Figure 11, Figure 12 and Figure 13, providing both values of ACC and area under the curve (AUC).

The ROC curves of the OCSVM approach are shown in Figure 8 and Figure 9. With WD=15 s, the worst performance was found in correspondence to changes in behavioral status AC-AP, AC-RE, AP-AC, AP-RE, RE-AC, RE-AP, with an ACC less than 80%. In the cases of RE-AC and RE-AP, the ACC was lower than 70%. In all other changes, the ACC was greater than 90%. Performance has significantly improved with WD = 70 s. Almost all state changes exhibited an ACC greater than 95%, except AC-AP and AC-RE settled at 94% and 93%, respectively.

As regards the BLSTM-AE approach, whose ROC curves aren’t provided in Figure 10 and Figure 11, in the case of WD=15 s, the worst performance was found for the state changes AG-RE, AC-AP, AP-RE, and RE-AC, with lower ACC values of 70%. The best performances, on the other hand, were found for changes of state AG-AP, AP-AG, and AP-AC. In the case of WD = 70 s, the performance was improved but not by that much. The BCP ACC of the AG-AP and RE-AC changes became even worse.

Finally, the ROC curves of the BLSTM-AE TCN approach are provided in Figure 12 and Figure 13. With this approach, even for WD = 15 s, the ACC of the BCP is higher than 96% for all state changes and it is better than all other approaches including the cases in which WD is equal to 70 s.

In this study, all presented network architectures were implemented and evaluated using the MathWorks^®^ Deep Learning Toolbox (v 14.2, R2021a, MathWorks Inc., Natick, MA, USA) [64] whereas the genetic optimizations were performed using the MathWorks^®^ Optimization Toolbox (v 9.1, R2021a, MathWorks Inc., Natick, MA, USA) [65].

For each observation window from 10 to 70 seconds, the experimentation was conducted through ten-fold cross-validation on the total number of samples (i.e., ranging from 12,636 samples for a 70-second windows to 14,796 samples for a 10-second windows).

The OCSVM approach was evaluated (trained and tested) on a computer system with CPU Intel^®^ Core™ i7-8565U at 2.00 GHz. The optimization of both the training and testing of the BLSTM-AE and TCN networks was performed on a computer system equipped with CPU Intel^®^ Core™ i7-5820 K at 3.30 GHz, and GPU NVIDIA GeForce^®^ GTX TITAN X. Finally, the optimization of both the training and testing of the joined network architecture BLSTM-AE TCN was performed on a computer system based on CPU Intel^®^ Core™ i9-10900 K 3.70 GHz, and GPU NVIDIA GeForce RTX™ 2060.

All the networks were trained from scratch using the Adam solver [66] with the gradient decay factor 0.9 and the initial learning rate 0.001. The number of epochs, instead, was different for the three networks, with 1000 epochs for BLSTM, 500 epochs for TCN, and 2000 epochs for BLSTM-AE TCN. The genetic optimization of the network hyperparameters was the process that took the most time, taking 35 days for BLSTM, 18 days for TCN, and 76 days for BLSTM-AE TCN.

## 4. Discussion

This study developed a new approach to predicting behavioral changes from neurovegetative parameters using learned features. The proposed approach was based on deep feature learning using a pretrained TCN. In particular, the pretraining process is based on non-field data, i.e., data not acquired by patients but prepared in a laboratory. This type of pretraining offers the advantage of making the system semi-supervised since patient data are only required during stimulation therapy to predict behavioral changes, carried out via the BLSTM-AE network.

The deep learning feature approach was then compared with the traditional approach based on handcrafted features, obtained through CATCH22, i.e., 22 features for each considered neurovegetative/physiological parameter (88 features for DS1 and DS2’, and 110 features for DS2) and classified via OCSVM. The comparison between handcrafted and learned features shows a higher processing time in the handcrafted case. This is due to the greater computational complexity of the CATCH22 extraction framework than the lighter TCN (testing phase) and the need to employ a wider observation window to obtain the same performance.

On the other hand, the handcrafted CATCH22 extraction process, although it may appear to be an automatic process, it is not entirely so. Indeed, in order to optimize the extraction process, it is recommended to make a feature selection to identify the most suitable set of features to exclude the least performing ones [67].

Furthermore, the learned feature approach based on joined BLSTM-AE and TCN exhibits shorter reaction times and better performance than OCSVM (handcrafted features based on CATCH22) and BLSTM-AE (learned feature) alone. As reported in Figure 6, the performances exhibited by the joint use of BLSTM-AE and TCN with a window of only 10 seconds can be bought with those provided by OCSVM and by BLSTM-AE alone with windows of 70 seconds. The architectures of the BLSTM-AE and TCN networks were specifically optimized to work together using the algorithm suggested in [61]. The performance was significantly lower without joint optimization, i.e., using the individually optimized BLSTM-AE network.

The experimentation was conducted with six (HR, RR, HRV, BP, GSR, ACT) and four (HR, RR, HRV, ACT) signals, demonstrating that more signals bring performance benefits. Furthermore, the results with four signals on patients and volunteers were quite comparable, confirming that the volunteers’ simulation of the behavioral states was carried out in a sufficiently realistic way.

Transitions involving the AC and AP states were more difficult to discriminate in the presence of four signals, particularly in the OCSVM and BLSTM-AE cases. In the OCSVM case (handcrafted features), AC–AP, AP–AC, and AC–RE transitions were more problematic (Figure 9). Instead, in the case of BLSTM-AE (learned features), the AG–AP, AG–RE, RE–AG, AC–AP, AP–AC, AC–RE, RE–AC, and AP–RE were more problematic (Figure 11). This may be partly because the AC state does not always manifest itself with body movements but often results in a state of sustained attention, making it difficult to discriminate from the AP state in the absence of the GSR neurovegetative signal.

The results achieved in the present study were compared with the state of the art. Given the absence of studies in the literature on predicting behavioral states such as those considered in this study, the comparison was conducted by considering studies aimed at detecting different stress levels. To this end, the scales of stress levels and behavioral states were placed alongside each other in consideration of four levels: Level −1, Level 0, Level 1, and Level 2. Level 0 indicates a total absence of stress, i.e., RE state. Level 1 corresponds to the healthy level of stress, eustress, which corresponds to the AC state considered in this study. Level 2, on the other hand, corresponds to excessive levels of stress, which also lead to a state of AG. In order to include the AP state, the negative level marked with −1 was introduced to indicate a state of no response, close to drowsiness, considered in some studies for the detection of stress and drowsiness while driving cars [22,24]. The comparison of the results achieved with the state-of-the-art is shown in Table 7. In the case of learned features, the ACC performances obtained in this study with 10-second windows with both four and six signals outperformed the current state of the art. In the case of handcrafted features, however, the performances outperformed the state of the art only in the presence of six signals with a window of 70 seconds. However, with shorter windows, the performances remain comparable to the state of the art.

Given the relevance of the window duration parameter, the performance was evaluated at different window duration values. On the one hand, to anticipate changes in the behavioral state, it is desirable to use windows of the shortest possible duration. On the other hand, very short windows do not guarantee optimal ACC performance, especially in the OCSVM and BLSTM-AE cases. In the case of the BLSTM-AE TCN approach, however, the dependence on the window duration is much less marked. This effect is presumably due to the overlap of two networks capable of handling time dependencies, namely the TCN network for feature extraction and the BLSTM-AE network for detecting behavioral changes.

As explained in detail by Diraco et al. [61], the genetic optimization process searches for an optimal combination of parameters within predetermined ranges of values. The genetic algorithm iteratively proceeds by manipulating the population of the candidate solutions to produce new solutions. Each candidate solution is a DNN architecture trained and tested at each iteration to update the fitness function. The search process continues until a Pareto-optimal solution is found, taking several thousand iterations. However, although the optimization process takes a long time, it is only performed once for a given combination of physiological parameters. One way to reduce the runtime of the optimization process is to consider subsampled physiological signals when optimizing DNN topologies. However, in this study, it was decided not to downsample the input signals, to approximate as much as possible the upper-performance limit of the investigated DNN architectures.

The main limitation of this study concerns the small number of patients involved, which, however, was extended by involving additional volunteers. A larger clinical trial involving more patients with dementia is currently underway.

## 5. Conclusions

The contribution of this study is three-fold: (1) a new approach for BSP based on BLSTM-AE TCN deep feature learning was presented; (2) feature learning (i.e., TCN) and change detection (BLSTM-AE) architectures were set up in order to operate jointly in an optimized way; and (3) the proposed framework was validated on four patients with dementia and five volunteers, using two datasets consisting of four (three neurophysiological and one of activity) and six signals (five neurophysiological and one of activity).

Although conducted on a small number of subjects, the validation demonstrated the feasibility of the BSP, which was subsequently incorporated into a CDSS within the MS-Lab project to support therapists during the administration of multisensory stimulation therapy.

Ongoing and future activities are focused on the clinical trial of the CDSS on a more statistically significant number of subjects with dementia. The data collected during the experimentation will be used to further evaluate the effectiveness of learned features to detect valuable indicators for predicting the patient’s behavioral state during therapy.

## Figures and Tables

**Table 1:** Overview of the collected datasets.

Dataset	Involved Subjects	Included Signals	Behavioral States
DS1	4 (patients)	HR, RR, HRV, ACT	AC, AG, AP, RE
DS2	5 (healthy volunteers)	HR,RR,HRV,BP,GSR,ACT	AC, AG, AP, RE
DS2’	5 (healthy volunteers)	HR, RR, HRV, ACT	AC, AG, AP, RE

**Table 2:** Time-series features suggested by Lubba et al. [28] and included in CATCH22 collection.

Type	Description
Distribution	Mode of z-scored distribution: 5-bin histogram.
	Mode of z-scored distribution: 10-bin histogram.
Simple temporal statistics	Longest period of consecutive values above the mean.
	Time intervals between successive extreme events above the mean.
	Time intervals between successive extreme events below the mean.
Linear autocorrelation	First 1/e crossing of autocorrelation function.
	First minimum of autocorrelation function.
	Total power in lowest fifth of frequencies in the Fourier power spectrum.
	Centroid of the Fourier power spectrum.
	Mean error from a rolling 3-sample mean forecasting.
Nonlinear autocorrelation	$\mathrm{Time}\text{-}\mathrm{reversibility}\;\mathrm{statistic},\;{\left(x_{t+1}-x_{t}\right)}^{3}{}_{t}$.
	Automutual information, m=2,τ=5.
	First minimum of the automutual information function.
Successive differences	Proportion of successive differences exceeding 0.04σ.
	Longest period of successive incremental decreases.
	Shannon entropy of two successive letters in equiprobable 3-letter symbolization.
	Change in correlation length after iterative differencing.
	Exponential fit to successive distances in 2-d embedding space.
Fluctuation Analysis	Proportion of slower timescale fluctuations that scale with DFA (50% sampling).
	Proportion of slower timescale fluctuations that scale with linearly rescaled range fits.
Others	Trace of covariance of transition matrix between symbols in 3-letter alphabet.
	Periodicity measure of Wang et al. [59].

**Table 3:** Optimized parameters of the network architecture shown in Figure 4.

Network Parameters	Optimized Values
$B_{1},F_{1}$	16, 500
$B_{2},F_{2},D_{2}$	256, 50, 0.7810
$B_{3},F_{3}$	16, 500

**Table 4:** Optimized parameters of the network architecture shown in Figure 5.

Network Parameters	Optimized Values
K	5
$N_{1},S_{1},D_{1}$	256, 8, 0.6116
$N_{2},S_{2},D_{2}$	256, 6, 0.6391
$N_{3},S_{3},D_{3}$	256, 19, 0.0438
$N_{4},S_{4},D_{4}$	256, 8, 0.6323
$N_{5},S_{5},D_{5}$	256, 7, 0.5121

**Table 5:** Optimized parameters of the network architecture shown in Figure 7.

Network Parameters	Optimized Values
$B_{1},F_{1},D_{1}$	256, 200, 0.0083
$B_{2},F_{2},D_{2}$	128, 100, 0.2875
$B_{3},F_{3},D_{3}$	256, 200, 0.0095

**Table 6:** Average ACC percentages of the three approaches for the varying window durations.

WD (seconds):		70 s	60 s	50 s	40 s	30 s	20 s	15 s	10 s
Method	Dataset								
OCSVM	DS1	95.90	94.19	91.98	87.80	85.36	82.28	79.03	NA
	DS2	98.24	97.66	96.51	93.26	91.31	88.79	85.68	NA
	DS2’	97.69	97.39	96.24	92.77	91.06	88.61	85.55	NA
BLSTM-AE	DS1	85.98	85.02	83.14	81.26	80.97	80.46	79.37	74.97
	DS2	91.01	87.78	86.82	86.61	85.69	85.14	84.93	83.92
	DS2’	86.36	86.09	85.93	85.75	84.17	83.75	83.05	82.55
BLSTM-AE TCN	DS1	99.25	98.92	98.91	98.72	98.71	98.69	98.68	98.44
	DS2	99.42	99.28	99.18	99.11	99.04	99.02	98.92	98.38
	DS2’	99.21	99.13	99.03	98.94	98.89	98.81	98.59	98.42

**Table 7:** Comparison of the achieved results with the state of the art.

Authors	Physiological Signals	Features	ACC (%)
Healey and Picard [18]	ECG, EMG, GSR, RA	Handcrafted	97.40
Zhang et al. [20]	EMG, GSR, HR, RA, BP	Handcrafted	90.53
Wang et al. [22]	HRV	Handcrafted	88.28
Chiang [23]	ECG, HRV	Handcrafted	95.10
Chen et al. [24]	ECG, EMG, GSR, RA	Handcrafted	89.70
Zhang et al. [25]	ECG, EMG, GSR	Handcrafted	92.36
Nigam et al. [50]	ECG, GSR, RA, BT, TA	Handcrafted	98.00
Zontone et al. [51]	ECG, GSR	Handcrafted	88.13
Wang and Guo [32]	ECG, GSR, HR, HRV, RA	Learned	90.09
Mou et al. [49]	EYE	Learned	95.50
This study	HR, RR, HRV, ACT	Handcrafted	79.03/95.90 *
This study	HR,RR,HRV,BP,GSR,ACT	Handcrafted	85.68/98.24 *
This study	HR, RR, HRV, ACT	Learned	98.44/99.25 *
This study	HR,RR,HRV,BP,GSR,ACT	Learned	98.38/99.42 *

* The double values indicate the ACC with a 10- and 70-second window, respectively.

## Nomenclature

AC	Active
ACC	Accuracy
ACT	Activity Level
AE	Autoencoder
AG	Agitated
ANS	Autonomic Nervous System
AP	Apathetic
ARIMA	Auto-Regressive Integrated Moving Average
AUC	Area Ander the Curve
BLSTM	Bidirectional Long Short-Term Memory
BLSTM-AE	Bidirectional Long Short-Term Memory Autoencoder
BP	Blood Pressure
BSC	Behavioral State Change
BT	Body Temperature
CATCH22	Canonical Time-Series Characteristics 22
CDSS	Clinical Decision Support System
DCC	Dilated Causal Convolution
DNN	Deep Neural Network
DS1	Dataset 1
DS2	Dataset 2
ECG	Electrocardiogram
EMG	Electromyogram
FN	False Negative
FP	False Positive
GSR	Galvanic Skin Response
HR	Heart Rate
HRV	Heart Rate Variability
LSTM	Long Short-Term Memory
MLP	Multi-Layer Perception
MMSE	Mini-Mental Statement Examination
MS-Lab	Multi Sensorial Stimulation Lab
OCSVM	One-Class Support Vector Machine
PCA	Principal Component Analysis
PNS	Parasympathetic Nervous System
RA	Respiration Amplitude
RE	Relaxed
ReLU	Rectified Linear Unit
RNN	Recurrent Neural Network
ROC	Receiver Operating Characteristic
RR	Respiration Rate
SBL	Sparse Bayesian Learning
SNS	Sympathetic Nervous System
SVD	Single Value Decomposition
TA	Triaxial Acceleration
TCN	Temporal Convolutional Network
TN	True Negative
TP	True Positive
WD	Window
